# From Text to Thought: How Analyzing Language Can Advance Psychological Science

**DOI:** 10.1177/17456916211004899

**Published:** 2021-10-04

**Authors:** Joshua Conrad Jackson, Joseph Watts, Johann-Mattis List, Curtis Puryear, Ryan Drabble, Kristen A. Lindquist

**Affiliations:** 1 Department of Psychology and Neuroscience, University of North Carolina at Chapel Hill; 2 Department of Linguistic and Cultural Evolution, Max Planck Institute for the Science of Human History; 3 Center for Research on Evolution, Belief, and Behaviour, University of Otago; 4 Religion Programme, University of Otago

**Keywords:** natural-language processing, comparative linguistics, historical linguistics, psycholinguistics, cultural evolution, emotion, religion, creativity

## Abstract

Humans have been using language for millennia but have only just begun to scratch the surface of what natural language can reveal about the mind. Here we propose that language offers a unique window into psychology. After briefly summarizing the legacy of language analyses in psychological science, we show how methodological advances have made these analyses more feasible and insightful than ever before. In particular, we describe how two forms of language analysis—natural-language processing and comparative linguistics—are contributing to how we understand topics as diverse as emotion, creativity, and religion and overcoming obstacles related to statistical power and culturally diverse samples. We summarize resources for learning both of these methods and highlight the best way to combine language analysis with more traditional psychological paradigms. Applying language analysis to large-scale and cross-cultural datasets promises to provide major breakthroughs in psychological science.

Humans have been using language for millennia and compiling written records for at least the past 5,000 years (Walker & Chadwick, 1990). In that time, humans have written nearly 130 million books (Tachyer, 2010), producing sprawling religious scriptures, millions of songs, countless speeches, and expansive dictionaries that explain and translate entire lexicons. These records of human language represent a rich but underexplored trove of data on the human experience.

Human language—be it spoken, written, or signed—has the power to reveal how humans organize thoughts into categories, view associations between these categories, and use these categories in daily life for communication and social influence. It can be used to understand how humans view the salience of different ideas and how understanding of these ideas may change over time. On a broader level, language can reveal variation in thought processes and verbal behavior across different cultural and ideological groups and illuminate universal and variable patterns in how humans understand constructs such as God, emotion, and the self. Language is thus a rich and dynamic window into human experience that promises to yield new insights in each branch of psychological science.

The promises of language analysis for psychological science were largely unrealized for most of the field’s history because most records of language were inaccessible. Books gathered dust on shelves, sacred texts lay in museums, and songs were stored either in human memory, on cassette tapes, or in albums. These vast stores of natural linguistic data sat out of reach over the 20th and early 21st centuries, and psychologists developed increasingly sophisticated measures of explicit attitudes (Likert, 1932), implicit attitudes (Greenwald et al., 1998), brain activity (Nichols & Holmes, 2002), and physiology (Kagan et al., 1987). But this is beginning to change.

Just as the printing press made language accessible to the masses, computational innovations are now making language analyzable for the academic masses. A methodological arms race in computational linguistics and computer science is producing new techniques that are capable not only of digitizing written language but also of efficiently processing, storing, and quantifying patterns in this language. As a result of these innovations, records of language are no longer hidden away but are freely and easily accessible. Researchers can now retrieve vast stores of digitized written text from thousands of languages around the world and throughout history and finally begin realizing the potential of language analysis for psychological science.

With newly developed databases and analytic tools, language analysis is trickling into psychological science. Here we discuss how psychologists can best leverage these tools to make predictions about human experience by explaining popular new methods of language analysis and psychological predictions that are suitable for these methods. We focus primarily on topics central to social psychology, such as emotion, religion, and creativity, but we also give examples from clinical, developmental, and cognitive psychology.

The main goal of this article is to provide a “one-stop shop” for psychological scientists to read about the history and best practices associated with different methods of language analysis and to provide resources for easily learning these methods. Although there are existing reviews of specific language-analysis methods (e.g., Bittermann & Fischer, 2018; Pennebaker et al., 2007; Rudkowsky et al., 2018) and some broader reviews about the utility of language analysis for the social and organizational sciences (e.g., Berger et al., 2020; Boyd & Schwartz, 2020; Kjell et al., 2019; Short et al., 2018), few articles have discussed how multiple forms of linguistic analysis can be integrated to address a range of psychological questions. We provide this information so that, as the trickle of text analysis in psychology becomes a flood, psychologists will be prepared to analyze language rigorously, accurately, and in a manner that takes full advantage of each method’s promise.

We also highlight systemic advantages of language analysis, focusing on the promise of natural-language processing (NLP) and comparative linguistics. NLP paradigms may be uniquely suited to resolve problems associated with the generalizability of psychological findings because they sample from real-life conversations, speeches, and texts and are useful for solving the problems associated with low statistical power because they often incorporate millions of datapoints (Bakker et al., 2016; Cohen, 1992). Comparative-linguistics paradigms may be uniquely suited to resolve problems of representation and diversity in psychology by incorporating traditionally underrepresented cultures (Chandler et al., 2019; Henrich et al., 2010; Rad et al., 2018). Language analysis is therefore well suited to address several of the largest current challenges in psychological science.

We suggest that language-analysis methods, because of their theoretical and practical advantages, are at least as valuable as Likert scales, measures of implicit bias, behavioral measures, neuroimaging, psychophysiology, and other paradigms in psychological science. We also review limitations of language analysis that make it well suited to complement (rather than replace) these existing methods. By complementing traditional methods with rigorous language analysis, we can gain a more complete understanding of the human mind.

## What Does It Mean to Analyze Language?

Humans are intuitive language analysts. Just as psychologists use measurements to index latent constructs, humans infer the latent meaning being conveyed via language. Humans recognize words, react to sentiment and affect in sentences, and search for meaning in metaphors and innuendos. Formal language analysis requires going beyond this intuition to quantitatively deconstruct the meaning of language and measure the constructs that it conveys. People may feel inspired when they hear a rousing speech, but how can the construct of “inspiration” be quantified by examining the length, content, and format of a sentence? Translation dictionaries may equate two words and report that they have the same meaning, but how can researchers test whether language speakers actually use these words to communicate the same ideas?

### The roots of language analysis in psychological science

Questions about how psychological meaning is embedded in language have deep roots in psychology, and many of the earliest psychologists were keenly aware of the promise of language analysis. Freud’s analytic techniques involved examining free associations and slips of the tongue (Freud, 1901). Murray’s Thematic Apperception Test analyzed the linguistic content of stories that people told in response to pictures (Murray, 1943), and Allport counted words in a dictionary to identify the structure of personality (Allport & Vernon, 1930). These early methods had substantial limitations and are rarely used in contemporary quantitative research, but they foreshadowed the impact of language analysis on psychological science.

The promise of language analysis for psychological theorizing was not fully realized until the development of computational methods of language analysis, the most popular of which may be the technique known as *linguistic inquiry and word count* (LIWC; Pennebaker et al., 2007; Tausczik & Pennebaker, 2010). LIWC uses word frequency to yield insight into the meaning of language. For example, words referencing social in-groups (e.g., “we,” “us”) are probably expressing more affiliative meaning than words referencing out-groups (“they,” “them”). LIWC uses these word-count methods with preprogrammed dictionaries that represent semantic categories and correspond to psychological constructs of interest. A negative-emotion dictionary counts a predetermined set of words that connote feelings of negative affect, whereas a pronouns dictionary counts instances of “she,” “I,” “they,” and other pronouns that can be used to assess whether someone is referring to the self or others. LIWC gives the percentage of words in a corpus that fall into each dictionary. This method has been generative in psychology, and studies have applied LIWC to understand the psychological effects of aging (Pennebaker & Stone, 2003), the content of lies (Newman et al., 2003), mental-health stressors such as bullying and domestic abuse (Holmes et al., 2007), political messaging (Gunsch et al., 2000; Pennebaker & Lay, 2002), the emotional toll of terrorist attacks (Back et al., 2010; Cohn et al., 2004), and the popularity of songs (Packard & Berger, 2020).

One of LIWC’s major strengths is its parsimony. The software takes corpora—stores of written text that have been structured in a way that makes them downloadable and analyzable by algorithms—and returns simple percentages summarizing the text’s content. But this strength is also a limitation. When analyzing a sentence with many positive words, counting alone cannot distinguish whether words are meant ironically or as part of a counterfactual statement, and it cannot determine the source or the target of this positivity. Consider, for example, an excerpt from Martin Luther King Jr.’s (1963) famous “I have a dream” speech:

We have also come to this hallowed spot to remind America of the fierce urgency of Now. This is no time to engage in the luxury of cooling off or to take the tranquilizing drug of gradualism. Now is the time to make real the promises of democracy. Now is the time to rise from the dark and desolate valley of segregation to the sunlit path of racial justice. Now is the time to lift our nation from the quicksands of racial injustice to the solid rock of brotherhood.

In just a few sentences, King’s speech uses the words “luxury,” “desolate,” “segregation,” and “justice.” A counting approach could identify themes of positivity, negativity, morality, and inequity, yet it would not identify the nuanced way that King intended these words to signal perseverance and a fight for progress. Many articles have pointed out the limitations of these “bag of words” approaches that simply count the number of words rather than examining how these words are used in context (Enríquez et al., 2016; Wallach, 2006). Some psychological paradigms have sought to address these gaps. For example, research on conceptual metaphors explores how words take on multiple meanings and how these can reflect psychological associations (e.g., the concepts “up” and “down” describe both physical placement and psychological mood; Crawford et al., 2006; Landau et al., 2010; Meier & Robinson, 2006). However, a drawback of conceptual-metaphor methods is that they qualitatively analyze language, making them difficult to apply to large-scale or cross-cultural datasets.

Another limitation of word-count methods is that they are focused almost exclusively on the English language, which limits their historical and cross-cultural generalizability. The English language (including Old English and Middle English) has existed for a small fraction of human history, and approximately 5% of people today speak English as a first language, yet English speakers probably account for more than 99% of language-analysis research published in psychology journals (Lewis, 2009). Some efforts have been made to translate LIWC to other languages, but these efforts are very recent and focus more on replication than on comparison (Windsor et al., 2019). This leaves open many questions about how seemingly equivalent words have different meanings across languages and whether more closely related languages have more similar meaning structures than more distantly related languages.

These limitations notwithstanding, word-count methods such as LIWC have been tremendously useful in psychology, and their limitations can be addressed by supplementing them with other methods of language analysis that are currently rarer in psychology. One of these traditions, NLP, uses methods developed in computer science to analyze semantic patterns in language. Another tradition, comparative linguistics, involves the comparison of languages to determine how languages have evolved over time, how they may communicate meaning in unique ways. Both methods were developed outside of psychology but have great potential for psychological research.

### NLP as a tool for studying large-scale patterns of cognition

#### Background

NLP—the interdisciplinary study of computer interaction with human language—is a relatively young area of study. NLP’s earliest notable paradigm was the “Turing Test”: the hypothetical test wherein a computer mimics human language so well that an observer cannot differentiate the computer from a real person (Turing, 1950/2009). Other early NLP developments involved ELIZA (Weizenbaum, 1966)—a computer therapist that could respond to human complaints (“I feel sad”) with realistic therapist comments (“and why do you feel sad?”)—and Jabberwacky.com, now running as “Cleverbot,” which was designed in the 1980s to simulate entertaining but realistic human conversations.

NLP was not necessarily designed with psychological insights in mind, but building algorithms to simulate human speech has obvious psychological implications. Many of these insights derive from the advancement of “machine learning”—computer algorithms that can improve automatically through experience. Machine learning approaches can either be *unsupervised*, in which algorithms such as topic models try to classify words without researchers providing feedback, or *supervised*, in which algorithms are trained on the evaluation and classification of data using feedback from researchers. For example, an unsupervised machine-learning algorithm could use a corpus of speeches to automatically identify major semantic themes on the basis of co-occurring words, whereas a supervised algorithm could be trained to recognize that negative words frequently precede positive words or even to recognize metaphors (Jacobs & Kinder, 2017). When applied to King’s speech, this algorithm would be able to do far more than a simple word-counting technique by potentially revealing themes of justice and liberty and identifying that metaphors such as a “sunlit path” are referring to morally commendable action.

Although early machine-learning approaches were limited by statistical methods and computational power, machine learning has taken huge steps in the past several decades. Early machine-learning models of language translation and production were built using constrained statistical methods (Weaver, 1955), rule-based methods (Nirenburg, 1989), and example-based methods (Nagao, 1984). These methods made simplistic assumptions about the cognitive processes underlying the production of language, such as the existence of a universal structure to grammar across languages. Today, artificial neural nets are at the forefront of research in machine learning and have more promise for actually understanding psychological processes. These networks are loosely modeled after the structure of organic brains by modeling associative networks of co-occurrence across many variables. Like the human brain, the way they process language can be complex and difficult to understand. But unlike the human brain, researchers can often ethically gain access to, and modify, the precise mechanisms underlying how these algorithms process language by delving into their code. This opens a new way of building and testing scientific theories within psychology (Cichy & Kaiser, 2019). In Figure S1 in the supplementary materials on OSF (https://osf.io/xycbd), for example, we describe a neural network that is designed to classify U.S. presidents’ speeches as being from either the pre-Civil War period or post-Civil War period to show how one of these algorithms can use text to make complex classification judgments.

NLP approaches now have a wide range of applications to psychological questions. These methods allow researchers to quantify the meaning of constructs in text or speech, identify the presence and extent of certain attitudes and emotions, and distill the meaning of words on the basis of how they are used in context. These algorithms can efficiently analyze millions of datapoints in seconds and have the potential to analyze more representative samples of subjects than typical undergraduate research pools or Mechanical Turk experiments, especially when they are applied to online blogs, diaries, or social-media websites such as Facebook or Twitter.

#### Application 1: quantifying the meaning of constructs

One of the most fundamental applications of NLP involves identifying the meaning of constructs and finding sets of constructs that cluster together in meaning. Topic modeling is a classic unsupervised NLP method that accomplishes this goal by finding co-occurring words that may represent psychological categories of interest. For example, a topic model might observe a construct such as “birthday” on the basis of the co-occurrence of such words as “happy,” “birthday” “cake,” “candle,” and “gift” (Hong & Davison, 2010; Wallach, 2006). Topic models can either match words to a predefined number of topics or freely extract the best-fitting number of topics from a set of texts using optimization.

Topic models each share a basic structure and output format, but they can be generated by different algorithms. One of these algorithms, latent semantic analysis, is arguably the most foundational method of generating topic models (Landauer et al., 1998), but it is not the only method. Probabilistic latent semantic analysis will include probabilities that words belong in topics (Steyvers & Griffiths, 2007); latent Dirichlet allocation is a Bayesian version of probabilistic latent semantic analysis (Blei et al., 2003), and structural topic models examine the relationships between variables and the prevalence of topics (Roberts et al., 2019). Researchers have used these kinds of topic models to estimate cross-cultural differences in people’s personal values (Wilson et al., 2016); predict the likelihood of clinical depression using people’s social-media updates (De Choudhury et al., 2013; Eichstaedt et al., 2018); quantify differences in the meaning of language across gender, age, and personality style (Schwartz et al., 2013); and estimate why some requests for favors are more effective than others (Althoff et al., 2014). A related set of models that classify texts (e.g., newspaper articles) rather than topics have helped match students’ reading level to their reading material (Graesser et al., 2011), identified differences in the thinking process of individuals with psychosis compared with control subjects (Elvevaag et al., 2010), and recognized different responses to a geopolitical event (Mishler et al., 2015).

Whereas topic models are focused on categorization, approaches involving word embedding quantify the meaning of concepts in a more continuous way; methods such as word2vec or GloVe (global vectors for word representation) map words or phrases to vectors of numbers using neural network models to create continuous numerical distances that represent differences in meaning (Goldberg & Levy, 2014; Mikolov et al., 2013). The semantic vectors produced by word embeddings allow researchers to map the meaning between any two concepts and to collect clusters of concepts that are the most similar to theoretically important “seed” concepts. For example, the seed concept of “freedom” might be closest in vector space to “autonomy” and relatively close to “choice” and “liberty.” These comparisons can help psychologists to measure and quantify otherwise abstract psychological constructs such as “freedom.” This approach has helped detect increasingly permissive culture in the United States via an increase in vocabulary related to “freedom” (Jackson, Gelfand, et al., 2019) and track the expanding concept of harm across the 20th and 21st centuries (Vylomova et al., 2019).

#### Application 2: tracking attitudes and emotions in unstructured data

Another NLP approach known as “sentiment analysis” goes beyond quantifying meaning and focuses on tracking attitudes and mood over time. Sentiment analysis is actually an umbrella term to capture a range of methods. “Knowledge-based” methods of sentiment analysis are similar to LIWC, insofar as they detect the frequency of different prespecified words and track how the frequency of these words changes over time (Caluori et al., 2020). For example, Cohn et al. (2004) tracked changes in affect after trauma, showing that positive-emotion language dropped sharply after the 9/11 terrorist attacks but then rebounded over time. Garcia and Rimé (2019) did a similar analysis of positive and negative collective emotions following the Paris terrorist attacks of 2015, and Vo and Collier (2013) used the approach to capture spikes in fear and anxiety following earthquakes. Hutto and Gilbert (2014) recently developed VADER (valence-aware dictionary and sentiment reasoner), a knowledge-based form of sentiment analysis that builds on LIWC by quantifying the intensity as well as the prevalence of positive and negative sentiment in text and incorporating slang into its dictionaries. VADER also uses several grammatical rules to detect preferences and emotions in nuanced contexts, such as when preferences are expressed through negations (“I do not dislike my partner”) or modifiers (“sometimes I really hate my friends”).

Combining grammatical rules with a human-validated lexicon (as VADER does) is a powerful and easily interpreted approach to sentiment analyses. Because the researcher specifies the set of rules ahead of time, there is no “black box” obscuring how the algorithm scores a segment of text. However, this strength is also its weakness. More complex tasks often benefit from learning which rules help to understand and classify text. Machine-learning methods, such as random forests and neural networks, are often better equipped to mine opinions in context because they can flexibly learn how patterns in input text (e.g., a smiley face) relate to some output (e.g., positive affect). Supervised approaches will often use a set of hand-labeled texts to train a sentiment classifier. Over the course of training, the model can learn how the presence of negation, emojis, or information from previous sentences help to correctly classify the text without requiring the researcher to explicitly implement any of these rules (Kiritchenko et al., 2014). For example, Wang and colleagues (2013) used a machine-learning approach to detect depression using the textual content of personal blogs with 80% accuracy, whereas Oscar and colleagues (2017) used a supervised machine-learning approach to capture stigma toward individuals with dementia.

#### Application 3: distilling linguistic information

**Fig. 1.**
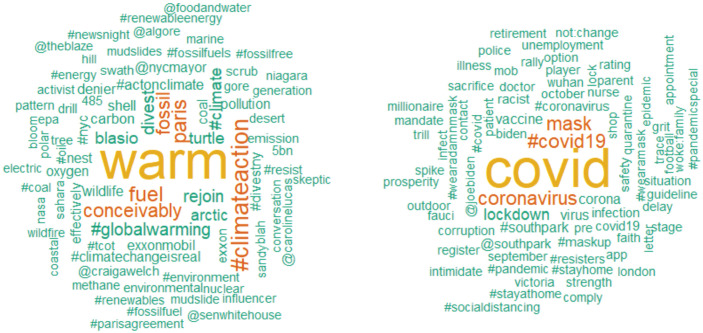
Words from tweets about climate change (left) and COVID-19 (right). These word clouds come from an algorithm called *term frequency-inverse document frequency* (TF-IDF), which is designed to highlight words that best distinguish between two corpora. This text was preprocessed using lemmatization and stop-word removal before visualization. Code for generating these plots is available in the supplementary materials on OSF (https://osf.io/hvcg3/).

A third set of NLP techniques is focused on more practical tasks, such as distilling and disambiguating the meaning of language as part of “preprocessing” text before additional analyses. These methods allow researchers to increase the signal in their data and reduce noise before testing hypotheses. For example, the method of lemmatization will remove inflectional endings to create a single form for words such as “walk,” “walking,” and “walked.” Sentence breaking will identify symbols such as periods or semicolons that demarcate semantic chunks. The emerging field of word-sense disambiguation uses context to disambiguate the true meaning of words that can be interpreted in different ways, such as the English word “funny” (Navigli, 2009). These preprocessing tools help distill language so that filler words are cut and words conveying important meaning are retained and made easier to detect. For example, Figure 1 shows a word-cloud of preprocessed keywords from tweets about climate change and tweets using COVID-19 hashtags. Note that there are no filler words such as “the” or “and” and that redundant forms of keywords (“ill” and “illness”) have been combined to minimize redundancy.

#### NLP resources

One distinct advantage of NLP algorithms is that they can operate over any sufficiently large digitally accessible corpora. In the early days of these algorithms, such corpora were difficult to find. But now there is a virtually limitless supply of digitalized text. As a case in point, the entire World Wide Web represents a digitalized corpus, and other corpora offer billions of words related to specific functions. The Google Books database contains a digitized corpus of books published in several languages over the past 400 years totaling more than 150 billion words (Michel et al., 2011). The Oxford English Corpus is the largest corpus of 21st century English, totaling more than 2.1 billion words across multiple English-language cultures (Oxford English Corpus, 2016). The *TIME Magazine* corpus of American English contains more than 100 million words of digitized *TIME Magazine* articles from 1923 to 2006 (Davies, 2007). The social-media sites Twitter (https://developer.twitter.com) and Reddit (https://www.reddit.com/dev/api/) both have easily accessible application programming interfaces (APIs), providing public access to millions of human interactions. Training NLP models can be an arduous task, and this training process benefits from large sources of data, but once models are trained, they can be easily applied to datasets of any size. Table 1 contains a list of corpora that were built for text analysis.

**Table 1.** Text Analysis Corpora

Corpus name	Link	Description
American National Corpus	http://www.anc.org/	Text corpus of American English containing 22 million words of spoken and written data since 1990. Mediums include email, tweet, and Web data, annotated for part of speech, lemma, and named entities.
British National Corpus	http://www.natcorp.ox.ac.uk/	Text corpus containing 100 million words of spoken and written language from the late 20th century from a variety of sources. Of the words, 90% are written and 10% are spoken. Tagged for parts of speech.
Corpus of Contemporary English	https://www.english-corpora.org/coca/	Text corpus containing 1 billion words of text from 1990 to 2019 from fiction, popular magazines, academic texts, TV and movie subtitles, blogs, and web pages. Allows searching by individual word. Tagged for parts of speech.
Google Books NGram Corpus	https://www.english-corpora.org/googlebooks/	Text corpus containing 200 billion words of written books. Subdivided into British English, American English, and Spanish. Mark Davies has made this corpus more accessible by allowing search by word, phrase, substring, lemma, part of speech, synonym, and collocates (nearby words). One strength of this corpus is its historical time span.
Oxford English Corpus	https://www.sketchengine.eu/oxford-english-corpus/	Text corpus of 21st-century English used by the makers of the Oxford English Dictionary, containing over 2 billion words. Includes language from many English-speaking countries and comprises many sources, including blogs, newspaper articles, emails, and social media. Tagged with extensive metadata. Users must apply for access through Oxford University Press.

NLP analyses may have been historically rare in psychology because they require advanced coding abilities. However, these barriers are now falling away as more psychologists develop proficiency with the R software environment (R Core Team, 2021). To help facilitate NLP proficiency in psychological science, we have created a five-part tutorial on NLP methods that covers (a) data acquisition and R packages, (b) preprocessing text data, (c) sentiment analysis using VADER, (d) word embeddings using GloVe, and (e) topic modeling. This R-based tutorial is available alongside our tutorial in comparative-linguistics methods in the supplementary materials at OSF (https://osf.io/hvcg3/).

### Comparative linguistics as a way to understand cultural diversity and universality

#### Background

Research on comparative linguistics—the study of similarities and differences between languages and the evolution of these characteristics—is far older than NLP but has been applied to psychological questions only recently. In the earliest days of the field, linguists such as the Danish scholar Rasmus Rask (1787–1832) and the German scholar Jacob Grimm (1785–1863) pointed to striking similarities between such geographically dispersed languages as Sanskrit, Gothic, Latin, and Greek (Geisler & List, 2013). Many of these early insights relied on the qualitative classification of cognates, defined as words or parts of words in different languages that trace back to common ancestral forms (Crystal, 2011). The word for the number 1, for instance, is a cognate that shares its basic form and sound across Indo-European languages such as English (“one”), French (“une”), and German (“eins”), suggesting that these languages evolved from a parent language that had a similar word for this number.

**Fig. 2.**
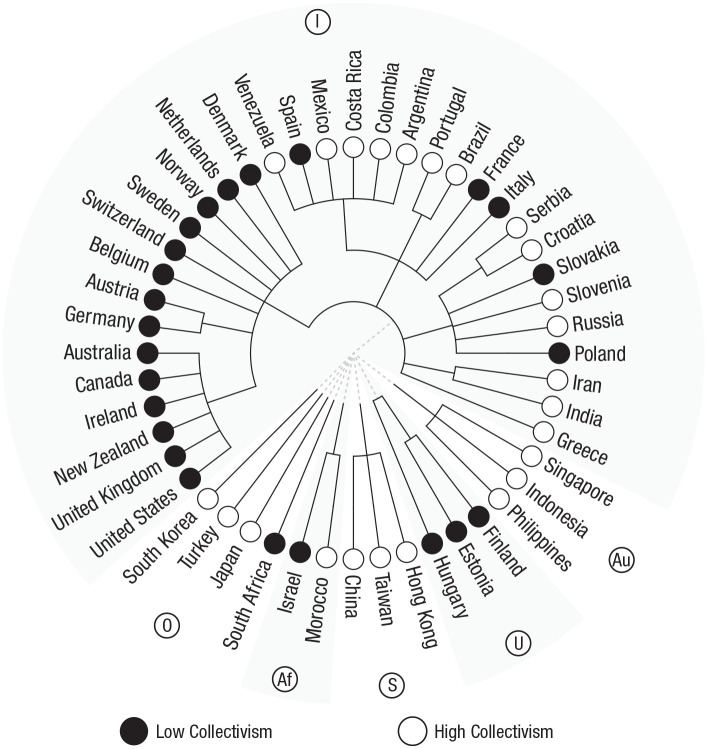
The global distribution of individualism and collectivism. Filled nodes represent individualist cultures (low collectivism; scores fall below the midpoint of the 1-to-100 scale from https://www.hofstede-insights.com/product/compare-countries/) and open nodes represent collectivist cultures (high collectivism; scores fall above the midpoint of the 1-to-100 scale). This distribution is represented on a language-based phylogeny. Cultures connected by solid lines are part of the same language family (language family data are from Bromham et al., 2018). The circled letters represent the following language families: I = Indo-European, Au = Austronesian, U = Uralic, S = Sino-Tibetan, Af = Afro-Asiatic, O = other.

Recent computational advances have expanded the scale and ambition of comparative linguistics. In particular, researchers have repurposed methods from biology to reconstruct language’s evolutionary ancestry. These approaches computationally aggregate many cognate classifications and use these classifications to develop language phylogenies (i.e., phylogenetic trees) that can be used to provide a proxy for cultural ancestry in the same way that biological phylogenetic trees display species’ ancestry. Figure 2 shows one such phylogenetic tree, in which modern countries are organized on the basis of the historical relationships between their predominant languages. This map shows that countries such as Singapore and Indonesia are “sister cultures” that share a more common ancestor than do Singapore and the United States. The center of Figure 2 represents a hypothetical common ancestor for all languages, which diverged and diversified as humans spread around the world.

Comparative-linguistics insights are interesting in their own right, but they also have a surprisingly wide range of application to psychological questions involving culture and psychology. Many of these applications rely on modeling the relationship between cultures, analyzing patterns of coevolution between cultural and behavioral factors, and comparing the meaning of constructs across languages. Computational comparative-linguistics approaches have also allowed for the compilation of huge databases of words and their associated meanings, which allows for cross-cultural comparisons on an unprecedented scale.

#### Application 1: modeling cultural interdependence

One of the most basic applications of comparative linguistics involves modeling interdependent datapoints in cross-cultural studies. Cross-cultural analyses will usually use regression to test for and explain patterns of variation across countries. These regressions assume that observations are independent, but comparative-linguistics research shows that many countries are interdependent because of their shared histories. Studies often treat Italy and Spain as independent units, for example, even though 80% of their lexicons overlap and the two societies share many features because of their recent common ancestry (Campbell, 2013). From a statistical standpoint, this is a case of “Galton’s problem”—interdependence between countries can lead to spurious correlations. For example, there is a highly cited link between cultures’ pathogen prevalence and political conservatism, which many scholars cite as evidence that disgust sensitivity makes people more conservative (Inbar et al., 2012). Yet this link is rendered nonsignificant when controlling for cultural and linguistic interdependence via cultures’ shared language families and geographic regions, suggesting that pathogen prevalence and political conservatism do not have a causal relationship (Bromham et al., 2018).

Fortunately, concerns about Galton’s problem can be partially alleviated by nesting cultures within their language families (Jackson et al., 2020). Modeling Indo-European as a group-level variable in a multilevel regression makes it less likely that a spurious association arises because of similarities between countries such as Italy and Spain. This kind of nested analysis is slowly becoming more common in cross-cultural research (e.g., Jackson et al., 2020; Skoggard et al., 2020) but it is still not standard practice in cross-cultural psychology.

#### Application 2: detecting patterns of cultural development

Cultural phylogenies also have the potential to yield important insights into the development of cultural differences because they track the relationship between linguistic and cultural groups over thousands of years. For example, consider worldwide variation in individualism–collectivism, which refers to cultures’ tendencies to either value individual rights and achievements (individualism) versus collective obligations and goals (collectivism). Most studies have observed that European countries are more individualistic than East Asian countries (Markus & Kitayama, 1991), but a cultural phylogeny can show that countries around the world with Germanic and Uralic languages are more consistently individualistic than countries with Latin and Slavic languages, suggesting that Northern and Central Europe may have historically been more individualistic than Western and Eastern Europe. In this way, phylogenetic trees can shed light on where and how cultural differences in human experience first emerged.

Whereas phylogenies represent the vertical inheritance of language and culture—where cultural information is passed down from one generation to another—it is also important to recognize that traits can be borrowed between groups, a process also known as horizontal transmission (Hoffer, 2002). For example, the word “honesty” in English is borrowed from the French language. Many comparative language databases flag suspected borrowings, and the World Loanword Database (WOLD; Haspelmath & Tadmor, 2009) is specifically designed to catalogue borrowings between languages. In principle, data on borrowings between languages could be represented in large-scale networks representing histories of contact and horizontal transmission between societies. Just as language phylogenies model the ancestry of cultures, language borrowing networks can model the diffusion of cultural constructs such as monogamy or psychological constructs such as intelligence. By tracking the diffusion of constructs through language, borrowing analyses have the potential to identify whether these factors are universal and, if they are not, why they have spread around the world over time. One plausible example could track whether the construct of self-esteem first emerged in individualist cultures in Western Europe and then was borrowed by collectivist cultures in South American and East Asia.

Modeling the evolutionary history of cultural variation also makes it possible to speculate about the causal origins of this variation. For the past decade, psychological science has begun grappling with the tremendous diversity in human culture and psychology, as well as the issues associated with focusing on WEIRD (Western, educated, industrialized, rich, and democratic) cultures (Henrich et al., 2010). Comparative-linguistics methods can not only analyze diverse samples but also examine sources of cultural diversity. For example, surveys published in *Science* and *Science Advances* have argued that rice farming (vs. wheat farming) is responsible for current-day cultural differences in collectivism (Talhelm et al., 2014, 2018), but these correlational surveys have not been able to causally test this hypothesis or even establish whether agricultural changes predated cultural changes. Using analyses that incorporate both phylogenetic trees and borrowing networks could help establish causal direction by testing between different models of coevolution between rice faming and collectivism (R. D. Gray & Watts, 2017).

Phylogenetic language trees can also yield insights about universal tendencies in how people change and transmit words, concepts, and behaviors over time. Many articles show that words for lower numbers are transmitted more reliably than words for higher numbers during the formation of new languages, perhaps because lower numbers are used more frequently than higher numbers (Pagel et al., 2007; Pagel & Meade, 2018). For example, the Latin word for the number 2, “duo,” has a similar sound and spelling to the French word “deux” and the Italian word “due,” but the Latin word “undeviginti,” meaning “19,” looks and sounds less similar to the French word “dix-neuf” and the Italian word “diciannove.” However, these studies have not yet considered how psychological variables could influence such cultural transmissions. On the other hand, psychological studies using the “Bartlett method”—in which statements are transmitted from person to person like a game of “telephone”—have uncovered several psychological transmission biases (Bartlett & Bartlett, 1932/1995). For example, high-arousal concepts are transmitted more reliably than low-arousal concepts, and social concepts are transmitted more reliably than asocial concepts (Mesoudi & Whiten, 2008), illustrating the salience of high-arousal feelings (Kensinger, 2004) and sociality (Cacioppo & Cacioppo, 2018) to human experience. Comparing results from this paradigm with rates of lexical evolution (the evolution of words) could assess whether concepts that are reliably transmitted in minutes-long social interactions are also reliably transmitted over thousands of years of history.

#### Application 3: quantifying cross-cultural differences in meaning

Comparative-linguistics methods are also well-suited to examine the meaning of emotions, moral values, personality traits, or other psychological factors across cultures by examining how these factors are expressed as linguistic *concepts* (meanings attached to words; Jackendoff, 1992). Insofar as language represents the psychological categories that are relevant to its speakers, it is a useful tool for psychologists to measure the extent to which a latent psychological *construct* (the latent meaning attached to clusters of observations; Fried, 2017) is shared within a culture over time or across cultures. For instance, researchers could examine how *concepts* such as “anger,” “disgust,” and “fear” are related to the psychological *construct* of emotion within or across languages.

One method for addressing this question examines a linguistic phenomenon called *colexification*, which occurs when two concepts are expressed with a single word (François, 2008; List et al., 2018). For example, the English word “funny” colexifies the concepts of “humorous” and “odd,” whereas the Russian word “ruka” colexifies “arm” and “hand.” As these examples illustrate, colexification often occurs when concepts are perceived as similar by speakers of a language (François, 2008), which makes frequency of colexification a useful measure of semantic closeness.

Studies are now beginning to build networks of colexifications to illustrate universality and cultural variation in semantic association across cultures. For example, Youn and colleagues (2016) showed that languages around the world had a similar meaning for physical entities such as “moon” and “sun” or “sea” and “lake,” suggesting that these concepts may have a universal meaning. Yet these colexification networks can also demonstrate cross-cultural variation if concepts show systematic variation in their colexifications across languages (Jackson, Watts, et al., 2019). For example, if “humorous” were colexified only with “odd” in European languages, this would suggest that strangeness is not a central aspect of humor across the world. Colexification is therefore a promising paradigm for testing whether Western theories about the universal structure of personality (e.g., “the big five”; Costa & McCrae, 2008), emotion (“basic emotions”; Ekman, 1999), morality (“moral foundations”; Graham et al., 2013), or psychopathology (American Psychiatric Association, 2013) generalize to non-Western cultures.

#### Comparative-linguistics resources

Comparative-linguistics resources are widely available, even though they are seldom used by psychologists. Many databases and datasets of comparative linguistics are publicly accessible and free to download. For example, the D-Place database contains language phylogenies representing the historical relationships among more than 1,000 human societies from around the world (Kirby et al., 2016), and the Database of Cross-Linguistic Colexifications (CLICS) contains colexifications from more than 2,000 languages (Rzymski et al., 2020). Other databases contain information on cross-cultural variation in grammar (Dryer & Haspelmath, 2013), word borrowing (Haspelmath & Tadmor, 2009), and vocabulary (Dellert et al., 2020) from a range of large and small languages. These databases provide rigorously vetted stimulus sets from enormous samples of cultures, and they often include data from small-scale cultural groups that are frequently underrepresented in psychological research. Table 2 summarizes several of these resources and provides links to their publicly available data.

**Table 2.** Public Datasets of Historical and Cross-Cultural Language

Database	Link	Description
D-Place	https://d-place.org/	Aggregates data on cultures’ evolutionary histories, ecologies, sociocultural structures, and geographic locations into one repository with rich metadata on sources of information, including previously established phylogenetic trees.
Cross-Linguistic Colexification Database	https://clics.clld.org/	Contains data on concept colexification from over 2,000 languages.
World Loanword Database	https://wold.clld.org/	Contains vocabularies of 1,000 to 2,000 entries for 41 languages around the world, as well as the likelihood that these words were borrowed from other languages.
Natural History of Song	https://osf.io/jmv3q/	Contains ethnographic descriptions of songs from 60 cultures. Also contains features of songs from 86 societies that were gathered through field recordings.
APiCS Online	https://apics-online.info/	A database of structural properties of creole and pidgin languages gathered from descriptive materials.
Glottolog	https://glottolog.org	A reference catalog of the worlds languages, providing expert classifications, geolocations, and references for more than 7,000 spoken and signed languages.
Concepticon	https://concepticon.clld.org	A reference catalog of concepts that are typically used in cross-linguistic studies, offering definitions, links to datasets in which the concepts were used, and additional metadata on psychological categories (norms, ratings, relations).
World Atlas of Language Structures	https://wals.info/	A large database of structural properties of language gathered from descriptive materials.

Note: Many of these databases are still in development, so their coverage will likely expand from these estimates.

Our supplementary materials at OSF (https://osf.io/hvcg3/) also contain tutorials for how to analyze phylogenetic trees (in R) and build colexification networks (in Python). These resources are intended for scholars with basic coding abilities but who have not yet used methods from comparative linguistics.

### Limitations and opportunities for language analysis

Language analysis has many advantages over traditional psychological methods, but it also comes with important limitations. Although NLP approaches offer an unprecedented scale of analysis, they will seldom be more accurate than a human coder. NLP techniques also carry the same gender and racial biases as the language- and human-generated labels they are trained upon (Garg et al., 2018; Kiritchenko & Mohammad, 2018). Preprocessing methods in NLP analysis also have a trade-off between parsimony and accuracy. An algorithm that removes stop words and lemmatizes key words will help make text analysis simpler, but it can also neglect important information in context. Words such as “warm” and “warming” may be lemmatized even though they have different implications for climate-change belief.

Comparative-linguistics methods face different challenges. One challenge to using language to study cultural variation is that language groups do not always neatly correspond to cultural groups. Cultural groups can speak multiple languages, and languages can span many cultures. A language phylogeny therefore provides only an approximation of how societies developed and diverged from one another and may not be appropriate when large-scale language replacement has occurred in a sample. Language phylogenies may also be biased by word borrowings. Language phylogenies are built from datasets that exclude known borrowings, but undetected borrowings can make two languages seem more similar than they really are (Greenhill et al., 2009). Finally, all language-analysis methods are limited by the fact that language is only a rough approximate of human experience.

The limitations of NLP and comparative-linguistics are not insurmountable. Methods of separating the likelihood of horizontal and vertical inheritance are growing more advanced (Atkinson et al., 2016; Sookias et al., 2018), and subsets of machine-learning classifications can be vetted by human coders to confirm their accuracy before interpretation. However, these limitations are important to acknowledge, and they make language analysis well suited to complement (rather than replace) other methods in psychology, such as experimental design, correlational surveys, neuroimaging, psychophysiology, and computational modeling.

Using different forms of language analysis together also combines their relative strengths. NLP and comparative linguistics were developed for different goals and in very different fields, and thus have mostly distinct strengths and weaknesses. Whereas NLP can analyze data on the scale of millions and with high granularity across time and person, comparative linguistics operates on a truly global scale and can make inferences about human culture long before the advent of writing. For this reason, these methods are a perfect match, and some articles are showing the potential of combining these methods. For example, one recent article on cultural differences in word meaning showed that semantic vectors in word embeddings correlated highly with colexification (Thompson et al., 2020), validating the two approaches and suggesting that long-standing patterns of meaning in language persist today.

**Fig. 3.**
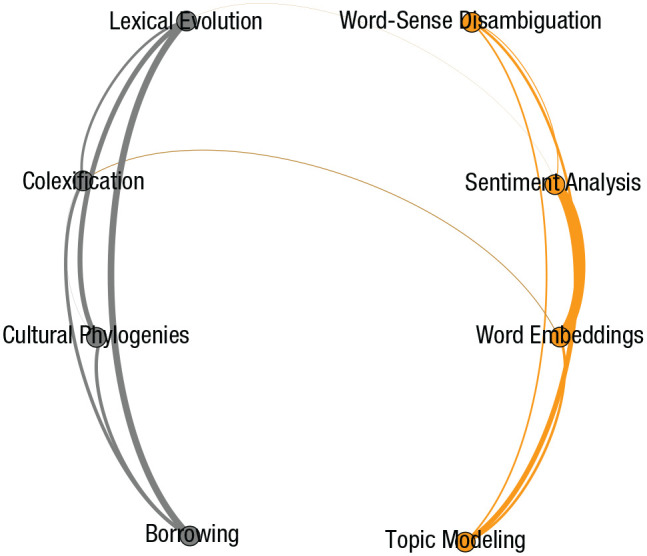
A bibliometric analysis of eight forms of language analysis. Each node is a method, and links between nodes represent first authors who have published using both methods. Colors are communities of clustering nodes from the community-detection algorithm infomap. This algorithm separated comparative-linguistics methods (in gray) and NLP methods (in orange), which have little cross-over but high within-cluster interconnectedness (i.e., researchers who use phylogenetic mapping also study borrowing but do not study word embeddings). Data come from Table S1 in the supplementary materials on OSF (https://osf.io/hvcg3/).

Unfortunately, researchers are rarely trained in both comparative linguistics and NLP. Figure 3 displays this dynamic in a network in which nodes represent methods and edge thickness represents the number of researchers who have been the first author on articles using different methods. The purpose of this figure, the data for which were drawn from a review of 200 different articles across NLP and comparative linguistics, is to underscore the lack of research that combines the scale of NLP with the cross-cultural and historical scope of comparative-linguistics methods. This network clearly shows that many researchers publish multiple methods within NLP and comparative linguistics, but few researchers publish methods that overlap both areas. Training in both sets of methods could foster interdisciplinary collaboration and increase the kinds of questions that scholars are able to answer.

## Applying Language Analysis in Psychological Science: Three Case Studies

**Fig. 4.**
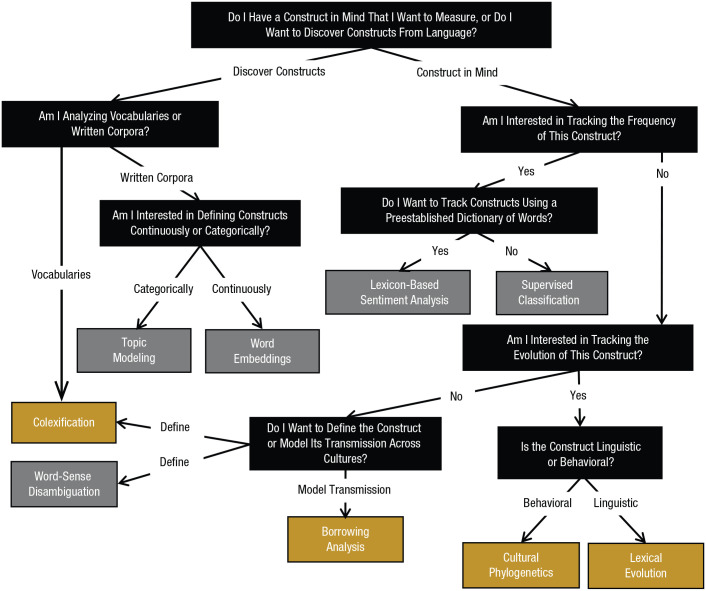
A flowchart of different language-analysis methods and the kinds of questions they are best suited to answer. Orange boxes represent methods from comparative linguistics, and gray boxes represent methods from NLP. Black boxes approximate the questions that may guide researchers toward these methods. Concepts are defined here as the meaning associated with words. This is meant as a general guide for researchers interested in language analysis, and there is some overlap in classifications. For example, word embeddings can show how language conveys moods and attitudes, and colexification can sometimes uncover evolutionary dynamics.

Psychological science still has work to do before researchers can master NLP and comparative linguistic methods. We dedicate the rest of this article to illustrating how that might happen. First, we present Figure 4, which is a visual flowchart illustrating how the language-analysis methods discussed in this article can be employed to address psychological questions. We then summarize three case studies that demonstrate how NLP and comparative linguistics can yield new insights and increase the scale and diversity of study into three psychological constructs that have been notoriously difficult to study—emotion, religion, and creativity. In these sections, we highlight research that has used language analysis to address new questions or solve long-standing debates or that has used language-analysis methods to increase the scale or cultural diversity of research in these fields. This work illustrates the utility of language analysis for asking enduring psychological questions and foreshadows the potential of these tools to address psychological constructs across social, cultural, cognitive, clinical, and developmental psychology.

### Emotion

Questions and debates about the nature of human emotion have existed since the earliest days of psychological science (Darwin, 1872/1998; James, 1884; Spencer, 1894; Wundt, 1897) and are relevant to psychological questions pertinent to social, clinical, and developmental psychology. Language-analysis methods have already increased the scope of this long-standing field and generated original methods of addressing old debates.

One of the most enduring debates about emotions concern whether emotions are universal, inborn categories that possess little variation around the world or are socially learned categories that vary in their experience and conceptualization across cultures (Cowen & Keltner, 2020; Ekman & Friesen, 1971; Izard, 2013; Plutchik, 1991; Lindquist et al., 2012; Mesquita et al., 2016; Russell, 2003). We recently addressed this question by means of a comparative-linguistics approach using colexifications (Jackson, Watts, et al., 2019). This analysis allowed us to increase the scale and generalizability over previous field studies of cross-cultural differences in emotion that had relied on smaller sample sizes and two-culture comparisons (Bryant & Barrett, 2008; Ekman & Friesen, 1971; Gendron et al., 2014, 2015, 2020).

**Fig. 5.**
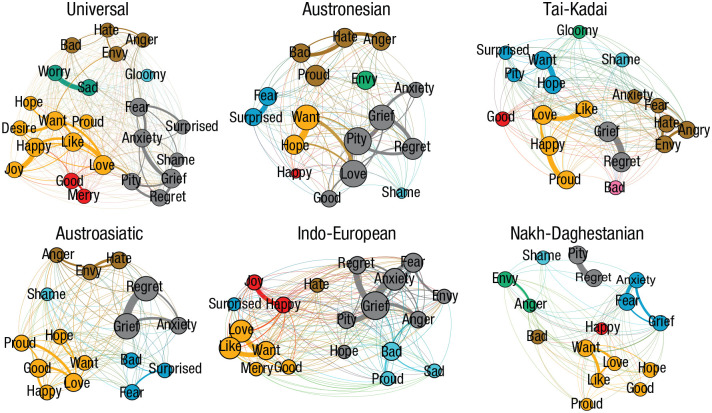
The colexification structure of emotion concepts for all languages (top left) and for five individual language families in Jackson and colleagues (2019) analysis of emotion. Nodes are emotion concepts, and links between concepts represent the likelihood that these concepts will be colexified in a language. Color indicates semantic community, which refers to clusters of emotions that are similar in meaning. From Jackson, J. C., Watts, J., Henry, T. R., List, J. M., Forkel, R., Mucha, P. J., Greenhill, S., Gray, R. D., & Lindquist, K. A. (2019). Emotion semantics show both cultural variation and universal structure. *Science*, *366*(6472), 1517–1522. https://doi.org/10.1126/science.aaw8160. Reprinted with permission from AAAS.

In our study, we computationally aggregated thousands of word-lists and translation dictionaries into a large database named “CLICS” (https://clics.clld.org/), and we used this database to examine colexification patterns of 24 emotion concepts across 2,474 languages. We constructed networks of colexification in which nodes represented concepts (e.g., “anger”) and edges represented colexifications (instances in which people had named two concepts with the same word), and then compared emotion colexification networks across language families. In contrast to Youn and colleagues (2016), who found universal colexification patterns involving concepts such as “sun” and “sky,” we found wide cultural variation in the colexification of emotion concepts such as “love” and “fear.” In fact, clusters of emotion colexification varied more than three times as much as the clustering patterns of colors—our set of control concepts—across language families (see Fig. 5). For example, “anxiety” was perceived as similar to “fear” among Tai-Kadai languages, but was more related to “grief” in Austroasiatic languages, suggesting that speakers of these language may conceptualize anxiety differently.

The variability in emotion meaning that we observed was associated with the geographic proximity of language families, suggesting that the meaning of emotion may be transmitted through historical patterns of contact (e.g., warfare, trade) and common ancestry. We also found that emotions universally clustered together on the basis of their hedonic valence (whether or not they were pleasant to experience) and to a lesser extent, by their physiological activation (whether or not they involved high levels of physiological arousal), suggesting valence and physiological activation might be biologically based factors that provide “minimal” universality to the meaning of emotion. In sum, this study used an unprecedented sample of cultures to yield new insights into the structure and cultural variation of human emotion.

A different set of language-analysis studies involving NLP are improving how psychologists measure emotion and track it over time and across social networks. For example, in a study of unprecedented historical scale, Morin and Acerbi (2017) used sentiment analysis to examine English fiction from 1800 to 2000 to assess whether the expression of emotion had changed systematically over time. They found a decrease in positive (but not negative) emotions conveyed in language over history in three separate corpora of text. This change could not be explained by changing writer demographics (e.g., age and gender), vocabulary size, or genre (fiction vs. nonfiction), raising the possibility that something about emotion or its expression has itself changed over time.

Other studies have also used language analysis to track faster emotional dynamics, such as measuring the emotional qualities of social-media posts (Roberts et al., 2012; Yu & Wang, 2015) and testing whether the emotions of one person are likely to rapidly spread via language throughout that person’s social network. Such studies have shown experimentally that emotional sentiment conveyed by language on social-media websites (e.g., Facebook) is more likely to make individuals who view that language express similar emotions (Kramer et al., 2014). Correlational studies find that social-media information with high emotional content is more likely to be shared than information with low emotional content (Brady et al., 2017). These studies show how affect can spread across many social-media users in a short period of time.

### Religion

The science of religion has a rich legacy equal to that of the psychology of emotion; many psychological studies have addressed questions about the social value and historical development of religion. Language analysis has recently begun answering both kinds of questions with a scope and ecological validity that was not possible with traditional methods.

NLP analyses have shed light on the positive and negative ways that religion affects happiness and intergroup relations. Some social theorists view religion as a primarily positive force because it reinforces social connections and promotes well-being (Brooks, 2007). On the other hand, “New Atheism” suggests that religion has a more negative effect on psychology by narrowing people’s worldviews and homogenizing the beliefs of religious adherents (Dawkins & Ward, 2006; Hitchens, 2008). Evidence for this debate has been mixed because of methodological challenges. For example, religious people frequently report more well-being than atheists in large national surveys, but they also show more social-desirability bias (Gervais & Norenzayan, 2012), which makes their self-reports less reliable.

NLP analyses are able to overcome these social-desirability limitations and have begun to show ecologically valid evidence that religion is linked to well-being. For example, Ritter et al. (2014) conducted a sentiment analysis of 16,000 users on Twitter and found that Christians expressed more positive emotion, less negative emotion, and more social connectedness than nonreligious users. Wallace et al. (2019) conducted a creative analysis of obituaries, finding that people whose obituaries mentioned religion had lived significantly longer than people whose obituaries did not mention religion, even controlling for demographic information.

Other NLP research has called the New Atheist proposition of religious worldview homogeneity into question. For example, Watts and colleagues (2020) analyzed the explanations that Christian and nonreligious participants generated to explain a wide range of supernatural and natural phenomena and estimated the overlap of these explanations as a measure of worldview homogeneity. If religion does indeed homogenize adherents’ worldviews, one would expect that religious people’s explanations would share greater overlap than nonreligious people’s explanations. Watts and colleagues (2020) used a text analysis approach known as Jaccard distances, which was able to estimate the similarity between participants’ explanations of the world using overlapping key words, and test whether religious people offered more homogeneous explanations than did nonreligious people. Using this algorithm, the researchers found that religious people’s explanations of supernatural phenomena were more homogeneous than nonreligious people’s explanations, but their explanations of natural phenomena (e.g., the prevalence of parasites) were more *diverse* than were nonreligious explanations, probably because they drew on supernatural as well as scientific concepts when explaining the natural world.

Comparative linguistics has mostly contributed to questions about how religion has developed over time across cultures. Many of these analyses have focused on the “supernatural monitoring hypothesis”: that watchful and punitive gods contributed to the evolution of social groups by increasing in-group prosociality and fostering large-scale cooperation (Johnson, 2016; Norenzayan et al., 2016). This idea is nearly a century old, arguably dating back to Durkheim (1912/2008), but most tests of the hypothesis have been correlational, and there is an ongoing debate about whether societies with large-scale cooperation tend to adopt moralistic religions or societies that adopt moralistic religions tend to be more cooperative (Whitehouse et al., 2019).

Researchers using comparative-linguistics methods recently addressed these debates by focusing on the development of religion in the Pacific Islands, where linguistic analyses have mapped out cultural phylogenies that can then be repurposed for cross-cultural research (R. D. Gray et al., 2009). Using these phylogenetic trees and implementing a method known as *Pagel’s discrete* (Pagel, 1999), Watts and colleagues (2015) inferred the probability that ancestor cultures had high levels of political complexity (indicating large-scale cooperation), the probability that they believed in supernatural punishment, and the probability that they worshiped moralizing high gods. Their results showed partial support for both sides of the debate about religion and cooperation. Broad supernatural punishment (e.g., punishment for violating taboos) tended to precede and facilitate political complexity. However, belief in watchful and punitive high gods (e.g., the Christian God) tended to occur only when societies were already politically complex.

Phylogenetic analyses have also shed light on the darker side of religious evolution, such as ritualized human sacrifice practices, which were common across the ancient world. According to the social-control hypothesis, ritual human sacrifice was used as a tool to help build and maintain social inequalities by demonstrating the power of leaders and instilling fear among subjugates. Yet evidence in support of this theory was based largely on individual case studies showing that higher classes often orchestrated ritual sacrifices (Carrasco, 1999; Turner & Turner, 1999). Watts and colleagues (2016) tested this prediction by examining patterns of ritual human sacrifice and social inequality across 93 Pacific societies that had been mapped onto an established language phylogeny (R. D. Gray et al., 2009). They found evidence that ritual human sacrifice often preceded, facilitated, and helped to sustain social inequalities, supporting the social-control hypothesis.

### Creativity

Compared with the psychology of emotion and religion, that of creativity has a shorter history in psychology. Most psychologists agree that creativity contributes to personal feelings of self-fulfillment and societal innovation (Pratt & Jeffcutt, 2009; Wright & Walton, 2003), but the field is still exploring the best ways to measure creativity as a psychological construct. More than a dozen creativity-measurement paradigms exist in psychology. One such measure asks participants to name multiple uses for common household items such as article clips and bricks (Guilford, 1950), whereas others require participants to think of creative marketing schemes (Lucas & Nordgren, 2015) or draw an alien from another planet (Ward, 1994). In each paradigm, responses are qualitatively scored on creativity by trained research assistants. Although these tasks are themselves quite creative, the coding process can be onerous, and it can take months to obtain creativity ratings for a small behavioral study. Because these measures require custom tasks and laboratory settings, they are also rarely suitable for analyzing real-world creative behavior.

Language analysis has only recently been applied to study creativity, but NLP techniques are already advancing the measurement of creativity with paradigms that can be applied to both individuals in a small study as well as millions of people around the world. One such paradigm is “forward flow” (K. Gray et al., 2019). Forward flow asks people to free associate concepts, much like classic psychoanalysis methods. But rather than qualitatively deconstructing these free associations, forward flow uses word embeddings to quantitatively analyze the extent that present thoughts diverge from past thoughts. For example, because “dog” and “cat” are frequently used together in large corpora, “dog” → “cat” would not represent as much divergence as “dog” → “fortress,” which are less frequently used together. Forward flow correlates with higher creativity scores on validated behavioral tasks such as the multiple uses task, and creative professionals such as actors, performance majors, and entrepreneurs score highly on forward flow (K. Gray et al., 2019). Forward flow in celebrities’ social-media posts can even predict their creative achievement (K. Gray et al., 2019). Forward flow may represent a rich and low-cost measure that could help capture creativity across people and societies.

Other NLP analyses have captured creativity in terms of divergences from normative language (e.g., Kuznetsova et al., 2013). Much like an unorthodox-looking alien, unorthodox patterns of language can signal creativity. However, it can be difficult to distinguish nonnormative and creative language (e.g., “metal to the pedal,” which is a reformulation of “pedal to the metal”) from nonnormative and nonsensical language (e.g., “the metal pedal to”). Berger and Packard (2018) developed a potential solution to this problem in a study of the music industry and used this method to test how creativity related to a product’s success. Their approach first used topic modeling to develop words that frequently appeared in different genres of music. For instance, words about bodies and movement were often featured in dance songs, whereas words about women and cars were often featured in country music songs. The study next quantified each song from the sample on its typicality according to how much it used language typical of its genre. Analyzing these trends found that songs that broke from tradition and featured atypical language performed better than songs featuring more typical language, offering some evidence that people prefer creative cultural products.

Recent language-analysis studies have already made a considerable impact on the study of creativity and show the potential of NLP for capturing and quantifying variability in creativity across people and products. Although no comparative-linguistics research has examined creativity, this subfield also has great potential for examining whether creativity varies in its structure across cultures and how creativity has evolved across history. Some historical analyses suggest that creativity has been highest during periods of societal looseness—periods with less rigid social norms and more openness (Jackson, Gelfand, et al., 2019). But this research was done on American culture, and it is not clear whether these findings would generalize around the world.

## Conclusion

Humans use language to express thoughts, convey emotions, and show biases. Researchers now have the tools to analyze and interpret this language, and here we encourage psychologists to use these tools to advance the field. Although research using language analysis is still young, it has already yielded major insights into emotion, religion, creativity, and many other processes. We have focused primarily on social, affective, and cultural psychology in this article given our own areas of expertise, but language-analysis methods are just as suitable for personality, clinical, developmental, and cognitive psychology. For example, many studies referenced in this article used language analysis to detect psychopathology or dementia and to help improve learning material in classrooms, which are core challenges in these other psychological subfields.

Our goal is not only to summarize the theoretical potential of language analysis but also to provide resources for psychological scientists who are interested in adopting language analysis. To this end, we encourage interested readers to browse Table S1, which contains 200 articles employing the methods we have summarized here. We also encourage readers to browse the resources in Tables 1 and 2, which are all publicly and freely accessible, and to visit our tutorials at https://osf.io/hvcg3/ to see how language-analysis techniques are implemented in R.

With the proper rigor and training, the use of language analysis has the power to transform psychological science. It also allows our field to analyze data on a previously unimaginable scale and survey indigenous and historical groups that have been underrepresented in past psychological research. When used with more traditional methods, language analysis promises an enriched and more globally representative study of human cognition and behavior.

## Transparency

*Action Editor:* June Gruber

*Editor:* Laura A. King
